# Diagnosis/Classification Criteria for Behcet's Disease

**DOI:** 10.1155/2012/607921

**Published:** 2011-09-27

**Authors:** Fereydoun Davatchi

**Affiliations:** Behcet's Unit, Rheumatology Research Center, Shariati Hospital, Tehran University of Medical Sciences, Tehran 14117, Iran

## Abstract

*Historical Background*. The ISG criteria for Behcet's, created in 1990, have excellent specificity, but lack sensitivity. The International Criteria for Behcet's Disease (ICBD) was created in 2006, as replacement to ISG. The aim of this study was to compare their performance. *ISG and ICBD Criteria*. For ISG oral aphthosis is mandatory. The presence of any two of the following (genital aphthosis, skin lesions, eye lesions, and positive pathergy test) will diagnose/classify the patient as BD. For ICBD, vascular lesions were added, while oral aphthosis is no more mandatory. Getting 3 or more points diagnose/classify the patient as BD (genital aphthosis 2 points, eye lesions 2 points, and the remaining each one point). *Performance and Comparison of ISG and ICBD*. Their sensitivity, specificity, and accuracy (percent agreement), were tested in three independent cohort of patients from Far-East (China), Middle-East (Iran), and Europe (Germany). The sensitivity for ISG was respectively 65.4%, 78.1%, 83.7% and for ICBD 87%, 98.2%, and 96.5%. The specificity for ISG was 99.2%, 98.8%, 89.5% and for ICBD 94.1%, 95.6%, and 73.7%. The accuracy for ISG was 74.2%, 85.5%, 85.5% and for ICBD 88.9%, 97.3%, and 89.5%. *Conclusion*. ICBD has better sensitivity, and accuracy than ISG.

## 1. Historical Background

Although Behcet's Disease (BD) is relatively a young disease (described in 1937), it has already 16 sets of diagnosis/classification criteria. The first of them was proposed by Curth in 1946, less than 10 years after the description of the disease [1]. It was followed by Hewitt et al. in 1969 [2], Mason and Barnes in 1969 [3], Hewitt et al. revised in 1971 [4], Japan in 1972 [5], Hubault and Hamza in 1974 [6], O'Duffy in 1974 [7], Chen in 1980 [8], Dilsen et al. in 1986 [9], Japan revised in 1988 [10], International Study Group (ISG) in 1990 [11], Iran in 1993 [12], Classification Tree in 1993 [13], Dilsen revised in 2000 [14], Korea in 2003 [15, 16], and the International Criteria for Behcet's Disease (ICBD) in 2006 [17–19]. 

The ISG criteria were created in 1990 to bring a consensus on one set of criteria by the collaboration of France, Iran, Japan, Tunisia, Turkey, UK, and USA. With the sensitivity of ISG criteria being low [12, 20–25], during the first International Workshop of Behcet's Disease in Kuhtai (Austria), it was decided to create an international team to evaluate the performance of ISG criteria and to compare it with the existing BD criteria and revise it if necessary. 

The ITR-ICBD team was founded in 2004 with the participation of 27 countries (Austria, Azerbaijan, China, Egypt, France, Germany, Greece, India, Iran, Iraq, Israel, Italy, Japan, Jordan, Libya, Morocco, Pakistan, Portugal, Russia, Saudi Arabia, Singapore, Spain, Taiwan, Thailand, Tunisia, Turkey, and USA). The International Criteria for Behcet's Disease (ICBD) were presented to the International Conference of Behcet's Disease in Lisbon (Portugal) in 2006. Originally it had two formats, like the Iranian criteria. Later, it was decided to keep only the traditional format [17–19]. The ICBD were presented to the 2007 World Congress of Dermatology in Argentina and to 2009 ACR congress of Rheumatology in the USA [19].

## 2. ISG and ICBD Criteria

The ISG criteria [11] use 5 items. Two items are mucous membrane manifestations. They are oral aphthosis (OA) and genital aphthosis (GA). The third item is skin manifestations, comprising pseudofolliculitis (PF) and erythema nodosum (EN). The forth item is ocular manifestations. They are anterior uveitis (AU), posterior uveitis (PU), and retinal vasculitis (RV). The fifth item is the presence of pathergy phenomenon (PP). It is detected by the pathergy test [26–30]. In ISG criteria, the presence of OA is mandatory. Two other items from the 4 remaining (GA, skin, eye, PP) are necessary to classify a patient as having BD. 

For the international criteria, the ICBD [17–19], vascular manifestations (VMs) have been added to the 5 items of ISG criteria, because they are one of the characteristics of BD, and were used in many criteria before the advent of ISG (Mason and Barnes, Hewitt, Hubault and Hamza, Dilsen, Japan revised, and Dilsen revised criteria). VM is defined as superficial phlebitis, deep vein thrombosis, large vein thrombosis, arterial thrombosis, and aneurysm. Therefore, ICBD use six items: OA, GA, skin (PF, EN), eye lesions (AU, PU, RV), VM, and PP. In the ICBD, genital aphthous lesions and eye lesions have more diagnostic value than the others. They get each 2 points. The other 4 items (OA, skin, VM, PP) get one point each. A patient has to get 3 or more points to be diagnosed/classified as having BD.

## 3. Performance and Comparison of ISG and ICBD

Many ways and methods can be used to evaluate the performance of a criteria set. The most common used are sensitivity, specificity, and accuracy. Other methods are the positive predictive value, the negative predictive value, the positive likelihood ratio, the negative likelihood ratio, the diagnostic odds ratio, and Youden's index [35–39].

*Sensitivity* is the number of BD patients correctly classified (diagnosed) by the criteria. It is expressed as percentage (number of diagnosed BD patients, divided by the total number of BD patients, and then multiplied by 100) [35]. The sensitivity of ISG in their cohort of 886 patients was 92% [11]. The 95% confidence interval (95% CI) was 90% to 93.6%. The sensitivity of ICBD in their cohort of 2556 BD patients was 96.1% (95% CI 95.3–96.8). By chi-square test the difference between the two sets of criteria is statistically significant (*χ* ^2^= 23.439, *P* < 0.001). The sensitivity of ISG in the ICBD cohort of patients was 82.4% (95% CI80.9–83.9).

It is important to look at the sensitivity of the two criteria in independent cohort of patients. Three studies validated the ICBD in their cohort of patients: Germany in 2008 [31], China in 2008 [32], and Iran in 2010 [33]. The sensitivity of ISG was, respectively, 83.7% (95% CI 74.3–90.1), 65.4% (95% CI 60.2–70.5), and 78.1% (95% CI 77–79.1). The sensitivity of ICBD was, respectively, 96.5% (95% CI 89.7–99.2), 87% (95% CI 82.8–90.2), and 98.2% (95% CI 97.8–98.5). 

Table 1 shows the sensitivity of ISG in different cohort of patients from different parts of world [11, 12, 17–24, 31–33].

*Specificity *is the number of non-BD patients, correctly recognized as not having BD. It is expressed as percentage (number of non-BD patients correctly recognized as not having BD, divided by the total number of non-BD patients, then multiplied by 100) [35]. The specificity of ISG criteria in their own cohort of patients was 97% (95% CI 90.8–99.3). However, the number of control patients was only 97, and all other control patients having oral aphthosis were discarded from the original cohort of control patients [34]. The specificity of ICBD in their cohort of patients was 88.7% (95% CI 86.8–90.4). The specificity of ISG and ICBD in Germany, China, and Iran was, respectively, 89.5%, 99.2% and 98.8% (ISG), and 73.7%, 94.1%, and 95.6% (ICBD).  Table 2 shows the specificity of different criteria in different studies.

*Accuracy *or percent agreement is the ability of the criteria to correctly recognize BD patients from the non-BD patients. It is also expressed by percentage (number of diagnosed BD patients + number of non-BD patients correctly recognized as not having BD, divided by the total number of BD patients + total number of non-BD patients, and then multiplied by 100) [35]. The accuracy of ISG in their own cohort of patients was 92% (95% CI 90.1–93.5). The accuracy of ICBD in their own cohort of patients was 93.8% (95% CI 93–94.5). The accuracy of ISG and ICBD in Germany, China, and Iran was, respectively, 85.5%, 7402% and 85.5% (ISG), and 89.5%, 88.9%, and 97.3% (ICBD).  Table 3 shows the accuracy of different criteria in different studies.

*Positive predictive value* (PPV) demonstrates the probability that the positive test be true positive. PPV is more influenced by specificity than sensitivity. A criteria set with 90% sensitivity and 90% specificity will have a PPV of 90. If sensitivity increases to 95, PPV will improve to 90.5%, while if specificity increases to 95%, PPV will improve to 94.8%. PPV is also greatly influenced by the prevalence of the disease. Taking the above example, the PPV remains the same (90) in a dedicated BD clinic, where 50% of patients have BD and 50% are controls (patients mimicking BD but are not true BD). In the general population, with a prevalence of 80 for 100,000 inhabitants, the PPV becomes only 0.72. Therefore the results calculated in a specific setting cannot be used in another setting [33]. The PPV was higher for ISG than ICBD criteria in the 3 independent set of patients; however, the difference was very small in the Iranian patients, only 2.8% (Table 4).

*Negative predictive value* (NPV) indicates the probability of a negative test to be a true negative. The NPV also is influenced by the prevalence of the disease. On the contrary of PPV, the NPV is more influenced by sensitivity than specificity. It is also highly influenced by the prevalence of the disease [33]. 

*Positive likelihood ratio* (PLR) demonstrates the odds of having the disease. If PLR is superior to 5, it means that the test is related to the disease. It is highly influenced by specificity, as is the PPV. It is why the PLR is much higher for ISG criteria than ICBD (Table 4). Higher PLR for ISG means that, if ISG is positive, the chance of having BD is very high, but unfortunately ISG was negative in around 18% of subjects, in the 3 independent sets (Table 1).

*Negative likelihood ratio* (NLR) shows the odds of not having the disease. It is highly influenced by the sensitivity, as for the NPV. It has therefore better values for ICBD than for ISG criteria (Table 4). The high NLR for ICBD means that, if ICBD are negative, there are little chances for the patient to have BD (only 2% error rate for the Iranian patients: Table 4).

*Diagnostic odds ratio* (DOR) is a new way to show how much a test is reliable, like combining the PLR and NLR results. If DOR is 1, it means the test (criteria) does not discriminate between the patient and the control. The power of discrimination increases with higher values of DOR. The DOR of ISG is 294 and of ICBD is 1185 in the Iranian patients, demonstrating the high discriminative power of ICBD over ISG (Table 4).

*Youden's index* (YI) is a rather old (1950) and simple calculation, combining the results of sensitivity and specificity, to show the performance of the diagnosis criteria. The result goes from zero to one. The more the result approaches 1, the higher the performance of the test is. The ideal is one, meaning a sensitivity and a specificity of 100%. A sensitivity and a specificity of 90% will give a YI of 0.8. The YI of ISG is inferior to ICBD in China and Iran (Table 4).

## 4. Conclusion

ICBD are the latest diagnosis/classification criteria, created by the participation of 27 countries from different parts of the world. The large number of Behcet's disease patients and control patients, from inside and outside of the Silk Road, assures the variability needed to create an international criteria that can work in any country with different ethnicities. The validation of the criteria in the Far East, Middle-East, and Europe demonstrates its validity.

## Figures and Tables

**Table 1:** Sensitivity of different classification/diagnosis criteria.

Study	Reference	Number	Criteria	Sensitivity	
				%	95% CI
ISG 1990	[11]	886	ISG	92	90.0–93.6
			ICBD	—	—
Iran 1993	[12, 13]	2069	ISG	86.2	84.6–87.6
			ICBD	—	—
China 1996	[20]	79	ISG	—	—
			ICBD	—	—
APLAR 1998	[21]	216	ISG	72.2	65.9–77.8
			ICBD	—	—
Russia 2000	[22]	105	ISG	79.8	71.2–86.6
			ICBD	—	—
USA 2000	[23]	164	ISG	75.6	68.4–81.6
			ICBD	—	—
India 2004	[24]	50	ISG	72	58.2–82.6
			ICBD	—	—
Singapore 2004	[24]	37	ISG	46	31.1–61.6
			ICBD	—	—
China 2004	[24]	98	ISG	81	71.6–87.3
			ICBD	—	—
Korea 2004	[24]	1454	ISG	58	55.4–60.5
			ICBD	—	—
Iran 2004	[24]	4900	ISG	82	80.9–83.0
			ICBD	—	—
ICBD 2006	[17–19]	2556	ISG	82.4	80.9–83.8
			ICBD	96.1	95.3–96.8
Germany 2008	[31]	86	ISG	83.7	74.3–90.1
			ICBD	96.5	89.7–99.2
China 2008	[32]	322	ISG	65.4	60.2–70.5
			ICBD	87	82.8–90.2
IRAN 2010	[33]	6128	ISG	78.1	77.0–79.1
			ICBD	98.2	97.8–98.5

95% CI: 95% confidence interval.

**Table 2:** Specificity of different classification/diagnosis criteria.

Study	Reference	Number	Criteria	Specificity	
				%	95% CI
ISG 1990	[11]	97	ISG	97.0	90.8–99.3
			ICBD	—	—
Iran 1993	[12, 13]	1540	ISG	97.5	96.6–98.2
			ICBD	—	—
China 1996	[20]	35	ISG	79.8	63.7–90.1
			ICBD	—	—
APLAR 1998	[21]	145	ISG	99.3	95.7–100
			ICBD	—	—
Russia 2000	[22]	233	ISG	99.8	98.0–100
			ICBD	—	—
ICBD 2006	[17–19]	1163	ISG	96.0	94.6–96.9
			ICBD	88.7	86.8–90.4
Germany 2008	[31]	38	ISG	89.5	75.1–96.3
			ICBD	73.7	57.8–85.1
China 2008	[32]	118	ISG	99.2	94.8–100
			ICBD	94.1	88.0–97.3
IRAN 2010	[33]	3400	ISG	98.8	98.4–99.1
			ICBD	95.6	94.8–96.2

95% CI: 95% confidence interval.

**Table 3:** Accuracy of different classification/diagnosis criteria.

Study	Reference	Number	Criteria	Accuracy	
				%	95% CI
ISG 1990	[11]	983	ISG	92	90.1–93.5
			ICBD	—	—
Iran 1993	[12, 13]	3609	ISG	91	90.0–91.9
			ICBD	—	—
China 1996	[20]	114	ISG	79.8	71.4–86.2
			ICBD	—	—
APLAR 1998	[21]	361	ISG	85.8	81.9–89.1
			ICBD	—	—
Russia 2000	[22]	338	ISG	89.8	86.2–92.7
			ICBD	—	—
ICBD 2006	[17–19]	3719	ISG	86.7	85.6–87.7
			ICBD	93.8	93.0–94.5
Germany 2008	[31]	124	ISG	85.5	78.1–90.7
			ICBD	89.5	82.7–93.8
China 2008	[32]	450	ISG	74.2	70.0–78.0
			ICBD	88.9	85.6–91.5
IRAN 2010	[33]	9528	ISG	85.5	84.8–86.2
			ICBD	97.3	97.0–97.6

95% CI: 95% confidence interval.

**Table 4:** Predictive value, likelihood ratio, diagnostic odds ratio, and Youden's index.

Study	Reference	Criteria	PPV	NPV	PLR	NLR	DOR	YI
ISG 1990	[11]	ISG	96.8	92.4	30.7	0.08	371.8	0.89
		ICBD	—	—	—	—	—	—
Iran 1993	[12, 13]	ISG	97.2	87.6	34.5	0.14	243.6	0.84
		ICBD	—	—	—	—	—	—
APLAR 1998	[21]	ISG	99.0	78.1	103.1	0.28	368.4	0.71
		ICBD	—	—	—	—	—	—
Russia 2000	[22]	ISG	99.7	83.2	399	0.20	1971	0.80
		ICBD	—	—	—	—	—	—
ICBD 2006	[17–19]	ISG	95.4	84.5	20.6	0.18	112.4	0.78
		ICBD	89.5	95.8	8.5	0.04	193.4	0.85
Germany 2008	[31]	ISG	88.8	84.6	7.97	0.18	43.8	0.73
		ICBD	78.6	95.5	3.67	0.05	77.3	0.70
China 2008	[32]	ISG	98.8	74.1	81.7	0.35	234.4	0.65
		ICBD	93.6	87.9	14.7	0.14	106.7	0.81
IRAN 2010	[33]	ISG	98.5	81.9	65.1	0.22	294	0.77
		ICBD	95.7	98.2	22.3	0.02	1185	0.94

PPV: positive predictive value, NPV: negative predictive value, PLR: positive likelihood ratio, NLR: negative likelihood ratio, DOR: diagnostic odds ratio, and YI: Youden's index.

## References

[1] Curth HO (1946). Recurrent genito-oral aphthosis with hypopion (Behcet’s syndrome). *Archives of Dermatology*.

[2] Hewitt J, Escande JP, Laurent PH, Perlemuter L (1969). Criteres de prevision du syndrome de Behcet. *Bulletin de la SocieteFrancaise de Dermatology et de Syphiligraphie*.

[3] Mason RM, Barnes CG (1969). Behçet’s syndrome with arthritis. *Annals of the Rheumatic Diseases*.

[4] Hewitt J, Escande JP, Manesse S (1971). Revision of the diagnostic criteria of Behcet’s syndrome. *La Presse Medicale*.

[5] Behcet’s Disease Research Committee of Japan (1974). Behcet’s disease guide to the diagnosis of Behcet’s disease (1972). *Japanese Journal of Ophthalmology*.

[6] Hubault A, Hamza M, de Sezeet al S (1974). La maladie de Behçet en 1974. *L’actualité Rhumatologique 15*.

[7] O’Duffy JD (1974). Critères proposés pour le diagnostique de la maladie de Behçet et notes therapeutiques. *Revue de Medecine*.

[8] Chen SP, Zhang X-Q (1980). Some special clinical manifestations of Behcet’s disease—report of illustrative cases and review of literature (author’s transl). *Chinese jJournal of Internal Medicine*.

[9] Dilsen N, Konice M, Aral O, Lehner T, Barnes CG (1986). Our diagnostic criteria of Behcet’s disease—an overview. *Recent Advances in Behcet’s Disease*.

[10] Mizushima Y (1988). Recent research into Behcet’s disease in Japan. *International Journal of Tissue Reactions*.

[11] International Study Group for Behcet’s Disease (1990). Criteria for diagnosis of Behcet’s disease. *Lancet*.

[12] Davatchi F, Shahram F, Akbarian M, Wechsler B, Godeau P (1993). Accuracy of existing diagnosis criteria for Behcet’s disease. *Behcet’s Disease*.

[13] Davatchi F, Shahram F, Akbarian M, Wechsler B, Godeau P (1993). Classification tree for the diagnosis of Behcet’s disease. *Behcet’s Disease*.

[14] Dilsen N, Bang D, Lee ES, Lee S (2000). About diagnostic criteria for Behcet’s disease: our new proposal. *Behcet’s Disease*.

[15] Chang HK, Kim SY (2003). Survey and validation of the criteria for Behcet’s disease recently used in Korea: a suggestion for modification of the international study group criteria. *Journal of Korean Medical Science*.

[16] Chang HK, Lee SS, Bai HJ (2004). Validation of the classification criteria commonly used in Korea and a modified set of preliminary criteria for Behçet’s disease: a multi-center study. *Clinical and Experimental Rheumatology*.

[17] International Team for the Revision of the International Criteria for Behcet’s Disease (2006). Evaluation of the International Criteria for Behcet’s disease (ICBD). *Clinical and Experimental Rheumatology*.

[18] International Team for the Revision of the International Criteria for Behcet’s Disease (2006). Revision of the International Criteria for Behcet’s Disease (ICBD). *Clinical and Experimental Rheumatology*.

[19] Davatchi F, Schirmer M, Zouboulis C, Assad-Khalil S, Calamia KT on behalf International Team for the Revision of the International Criteria for Behcet’s Disease,“Evaluation and Revision of the International Study Group Criteria for Behçet’s Disease”.

[20] Dong Y, Yao Q, Wang M Behcet’s disease in China.

[21] APLAR subcommittee for Behcet’s Disease (1998). APLAR evaluation of Behcet’s disease diagnosis criteria. *APLAR Journal of Rheumatology*.

[22] Prokaeva T, Alekberova Z, Reshetnjak T, Bang D, Lee E, Lee S (2000). Evaluation of Behcet’s disease diagnosis criteria: study from Russia. *Behcet’s Disease*.

[23] Calamia KT, Davatchi F, Bang D, Lee E, Lee S (2000). Sensitivity of diagnosis criteria in United States patients with Behcet’s disease. *Behcet’s Disease*.

[24] Davatchi F, Shahram F, Kumar A, Cheng YK, Cheong CT, Bendrups A (2004). Comparative analysis of Behcet’s disease in the APLAR region. *APLAR Journal of Rheumatology*.

[25] Davatchi F, Shahram F, Akbarian M (1997). Behcet’s disease-analysisof 3443 cases. *APLAR Journal of Rheumatology*.

[26] Mansoori P, Chams C, Davatchi F, Nasution AR, Darmawan J, Isbagio H (1992). Pathergy phenomenon in Behcet’s disease, new aspects. *Rheumatology APLAR 1992*.

[27] Chams-Davatchi C, Davatchi F, Shahram F, Hamza M (1992). Longitudinal study of the Pathergy phenomenon in Behcet’s disease. *Behcet’s Disease*.

[28] Davatchi F, Chams-Davatchi C, Shahram F (2007). Pathergy test in Behcet’s disease: Change in incidence over the time. *APLAR Journal of Rheumatology*.

[29] Davatchi F, Chams-Davatchi C, Ghodsi Z (2011). Diagnostic value of pathergy test in Behcet’s disease according to the change in incidence over the time. *Clinical Rheumatology*.

[30] Davatchi F, Shahram F, Chams-Davatchi C (2010). How to deal with Behcet’s disease in daily practice. *International Journal of Rheumatic Diseases*.

[31] Altenburg A, Bonitsis NG, Papoutsis N, Pasak M, Krause L, Zouboulis CC (2008). Evaluation of diagnostic criteria including ICBD (2006) in Adamantiades-Behcet’s disease patients in Germany. *Clinical Experimental Rheumatology*.

[32] Zhang Z, Zhou W, Hao Y, Wang Y, Dong Y (2008). Validation of the International criteria for Behcet’s disease (ICBD) in China. *Clinical Experimental Rheumatology*.

[33] Davatchi F, Sadeghi Abdollahi B, Shahram F (2010). Validation of the international criteria for Behcet’s dDisease in Iran. *International Journal of Rheumatic Disases*.

[34] O’Duffy JD, Kokmen E, International Study Group for Behcet’s Disease (1991). Evaluation of diagnostic (“Classification”) criteria in Behcet’s disease: toward internationally agreed criteria. *Behcet’s Disease Basic and Clinical Aspects*.

[35] Szklo M, Neto FJ (2007). *Epidemiology, Beyond the Basics*.

[36] Kirshwood BR, Sterne JASC (2003). *Essential Medical Statistics*.

[37] Glas AS, Lijmer JG, Prins MH, Bonsel GJ, Bossuyt PMM (2003). The diagnostic odds ratio: a single indicator of test performance. *Journal of Clinical Epidemiology*.

[38] Kraemer HC (2004). Reconsidering the odds ratio as a measure of 2 × 2 association in a population. *Statistics in Medicine*.

[39] Youden WJ (1950). Index for rating diagnostic tests. *Cancer*.

